# Nup358 interacts with Dishevelled and aPKC to regulate neuronal polarity

**DOI:** 10.1242/bio.20135363

**Published:** 2013-10-16

**Authors:** Pankhuri Vyas, Aditi Singh, Prayag Murawala, Jomon Joseph

**Affiliations:** National Centre for Cell Science, Ganeshkhind, Pune 411007, Maharashtra, India; ‡ Present address: Technische Universität Dresden, DFG Center for Regenerative Therapies, Fetscherstrasse 105, Dresden 01307, Germany

**Keywords:** Polarity, Nucleoporin, Neuron, Nup358, Dishevelled, aPKC

## Abstract

Par polarity complex, consisting of Par3, Par6, and aPKC, plays a conserved role in the establishment and maintenance of polarization in diverse cellular contexts. Recent reports suggest that Dishevelled (Dvl), a cytoplasmic mediator of Wnt signalling, interacts with atypical protein kinase C and regulates its activity during neuronal differentiation and directed cell migration. Here we show that Nup358 (also called RanBP2), a nucleoporin previously implicated in polarity during directed cell migration, interacts with Dishevelled and aPKC through its N-terminal region (BPN) and regulates axon–dendrite differentiation of cultured hippocampal neurons. Depletion of endogenous Nup358 leads to generation of multiple axons, whereas overexpression of BPN abrogates the process of axon formation. Moreover, siRNA-mediated knockdown of Dvl or inhibition of aPKC by a pseudosubstrate inhibitor significantly reverses the multiple axon phenotype produced by Nup358 depletion. Collectively, these data suggest that Nup358 plays an important role in regulating neuronal polarization upstream to Dvl and aPKC.

## Introduction

Cell polarity is fundamental to many aspects of growth, development and homeostasis of multicellular organisms. A set of genes that were identified to be responsible for the asymmetric zygotic division of *Caenorhabditis elegans*, namely, *par1* to *par6* and an atypical protein kinase C (Kemphues, 2000; Kemphues et al., 1988), were subsequently found to be highly conserved players regulating the process of polarization from worms to mammals (Goldstein and Macara, 2007). The polarity proteins distribute to specific subcellular locations in diverse cellular contexts such as in one-cell embryo of *C. elegans*, Drosophila neuroblasts, polarized epithelial cells, migrating cells and differentiating neurons, and play a crucial role in generating and maintaining polarity (Arimura and Kaibuchi, 2007; Goldstein and Macara, 2007; Knoblich, 2008; Suzuki and Ohno, 2006).

The Par3-Par6-aPKC complex (Par polarity complex), a sub set of the Par proteins, specifically localizes to the anterior end of the dividing *C. elegans* one-cell embryos (Etemad-Moghadam et al., 1995; Tabuse et al., 1998), apical side of polarized epithelia (Suzuki et al., 2001) and leading edge of migrating cells (Etienne-Manneville and Hall, 2001). Such an asymmetric distribution of these key players is important in generating distinct cellular domains and achieving polarization, mostly through regulation of downstream effectors by aPKC-mediated phosphorylation (Goldstein and Macara, 2007). The key events modulated by Par proteins involve cytoskeleton arrangement and membrane trafficking (Harris and Tepass, 2010; Munro, 2006; Shivas et al., 2010).

A few signalling pathways have been shown to regulate the activity of the Par polarity complex, which include Cdc42 (Joberty et al., 2000; Lin et al., 2000), Ras (Yoshimura et al., 2006), Rap1B (Schwamborn and Püschel, 2004), and phosphoinositide signalling (Jiang et al., 2005; Ménager et al., 2004; Shi et al., 2003; von Stein et al., 2005). In epithelial cells, Cdc42 determines the localization of Par complex to the tight junctions through interaction with Par6 and activation of aPKC (Goldstein and Macara, 2007; Joberty et al., 2000; Lin et al., 2000). Activated Cdc42 also recruits the Par complex to the leading edges of migrating cells (Etienne-Manneville and Hall, 2001). It is believed that this results in the spatial inactivation of GSK3β, which promotes the interaction between microtubules and the plus end binding protein adenomatous polyposis coli (APC), leading to subsequent cortical capture and stabilization of microtubules in the direction of migration (Etienne-Manneville and Hall, 2003).

Neuronal cells are one of the highly polarized cell types. Par complex has been reported to be present at the tip of the growing axons (Shi et al., 2003), and the complex is transported to the location through direct interaction of Par3 with the KIF3A subunit of the Kinesin-2 complex, and APC (Nishimura et al., 2004; Shi et al., 2004). In a recent study, it was shown that the non-canonical Wnt signalling induces Dvl-mediated activation of aPKC to regulate neuronal polarization (Zhang et al., 2007). Such a conserved function of Wnt has also been identified during directed migration of astrocytes (Schlessinger et al., 2007).

We previously reported that the nucleoporin Nup358 interacts with APC, and regulates the process of polarized cell migration (Murawala et al., 2009). We wished to investigate whether Nup358 plays a conserved role in other contexts of cell polarization and to unravel the molecular mechanism involved. To address these questions, we chose to study the well-established model of axon–dendrite polarization of cultured rat hippocampal neurons.

Our findings showed that Nup358 interacts with Dvl and aPKC, and is indispensable for the process of axon specification. Removal of Nup358 from developing neurons leads to improper differentiation and generation of multiple axons, while overexpression of a Nup358 fragment important for interaction with Dvl and aPKC leads to significant abrogation of the process of differentiation. This effect of Nup358 is mediated through inhibition of aPKC activity, since the multiple axon formation phenotype caused by Nup358 depletion could be significantly reversed by inhibition of aPKC.

## Results

### Nup358 interacts with Dishevelled

**Fig. 1.**
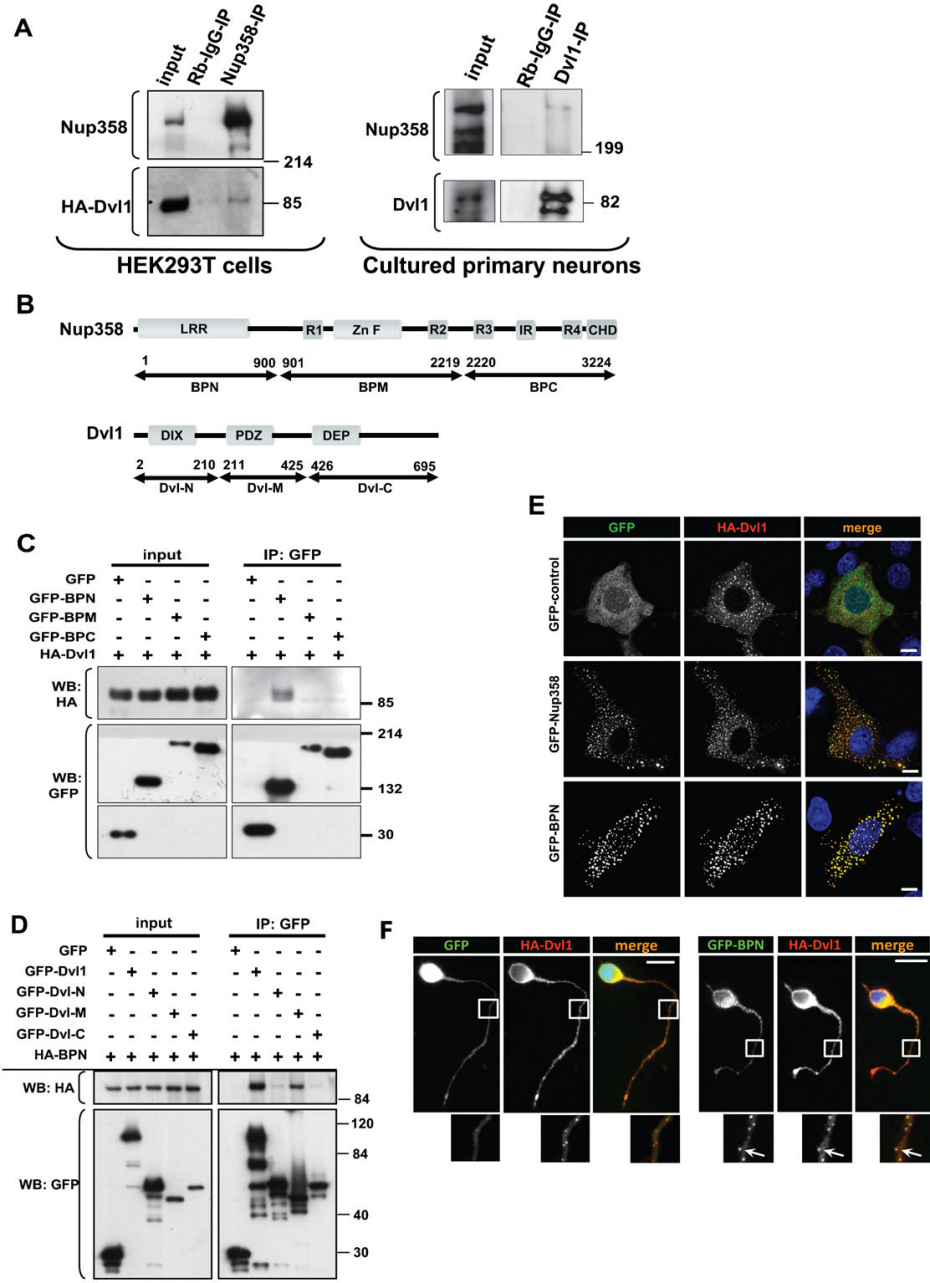
Nup358 interacts with Dishevelled. (A) *Left panel:* HA-tagged Dvl1 was overexpressed in HEK293T cells and endogenous Nup358 was immunoprecipitated (IP) using rabbit polyclonal antibodies against Nup358 (Nup358-IP). Rabbit IgG (Rb-IgG-IP) was used as control. Immunoprecipitates were analysed by western blotting (WB) with antibodies against HA and mAb414 (mouse monoclonal antibody that recognizes nucleoporins possessing FxFG sequences such as Nup358). Molecular weight markers are as indicated (in kDa). *Right panel:* Cultured primary neurons isolated from E18 rat were subjected to immunoprecipitation using Dvl1 antibody. The immunoprecipitate was probed with Dvl1 and Nup358 antibodies. (B) Domain structure of full-length Nup358 and Dvl1 and their fragments used in the study. The numbers refer to the amino acid positions. (C) HEK293T cells co-expressing HA-Dvl1 and GFP-control or GFP-tagged fragments of Nup358 were subjected to immunoprecipitation using anti-GFP antibodies. The immunoprecipitates were probed with antibodies against GFP or HA. (D) GFP-tagged full-length or fragments of Dvl1 were co-expressed with HA-BPN and were immunoprecipitated using anti-GFP antibodies. The immunoprecipitates were subjected to western analysis using indicated antibodies. (E) COS-7 cells were co-transfected with HA-Dvl1 (red) and GFP control, GFP-Nup358 or GFP-BPN (green) and were immunostained with anti-HA antibodies. DNA was visualized by Hoechst 33342 staining (blue). Scale bar, 10 µm. (F) E18 rat hippocampal neurons were transfected with pBetaActin-eGFP (GFP, green) or pBetaActin-BPN-eGFP (GFP-BPN, green) and pBetaActin-HA-Dvl1 (HA-Dvl1, red) constructs for 72 hours. The cells were fixed, stained and analyzed by fluorescence microscopy. DNA was stained with Hoechst 33342 dye. Scale bar, 20 µm. Arrows indicate co-localization of GFP-BPN with HA-Dvl1 puncta in neuronal extensions.

We previously reported that Nup358 interacts with and regulates the localization of APC, a component of Wnt signalling pathway, during polarized cell migration (Murawala et al., 2009). Immunoprecipitation analysis of HEK293T cells transfected with HA-tagged Dvl1 suggested a physical interaction between Nup358 and Dvl *in vivo* (Fig. 1A). Consistent with the interaction, GFP-tagged Nup358 and HA-tagged Dvl1 were found to co-localize into cytoplasmic puncta (Fig. 1E). These results suggest that Nup358 and Dvl exist in a complex *in vivo*.

Next, the region of Nup358 important for interaction with Dvl was mapped using cells expressing HA-Dvl1 and GFP-tagged fragments of Nup358: namely, N-terminus (spanning 1–900 amino acids, BPN), middle region (901–2219 amino acids, BPM) or C terminus (2220–3224 amino acids, BPC) (Joseph and Dasso, 2008) (Fig. 1B). Immunoprecipitation results suggested that Dvl1 associated specifically with the N-terminal region (BPN) of Nup358 (Fig. 1C). In agreement with the interaction data, both Dvl1 and BPN co-localized into cytoplasmic puncta in COS-7 cells (Fig. 1E) and neurons (Fig. 1F, arrow). However, as reported earlier, independent expression of BPN results in its localization to interphase microtubules (Joseph and Dasso, 2008) (supplementary material Fig. S1B). Co-immunoprecipitation experiments further suggested that the middle region of Dvl1, encompassing the PDZ domain, was involved in the interaction with Nup358 (Fig. 1D).

Consistent with the involvement of PDZ domain in the interaction with Nup358, Dvl2 deletion mutant that lacks the PDZ domain (Dvl2ΔPDZ) failed to interact with BPN (supplementary material Fig. S1A). However, under the same conditions, a mutant of Dvl2 that is devoid of the N-terminal DIX domain involved in oligomerization (Schwarz-Romond, 2007) (Dvl2ΔDIX) retained the interaction with BPN. Moreover, COS-7 cells expressing Dvl2ΔPDZ or Dvl1ΔPDZ and BPN failed to co-localize with each other. Under these conditions, BPN localized to microtubules and DvlΔPDZ mutants showed a diffused staining in the cytoplasm (supplementary material Fig. S1B). Collectively, these results indicate that the interaction between Nup358 and Dvl could be mediated through the PDZ domain.

### Nup358 associates with aPKC

**Fig. 2.**
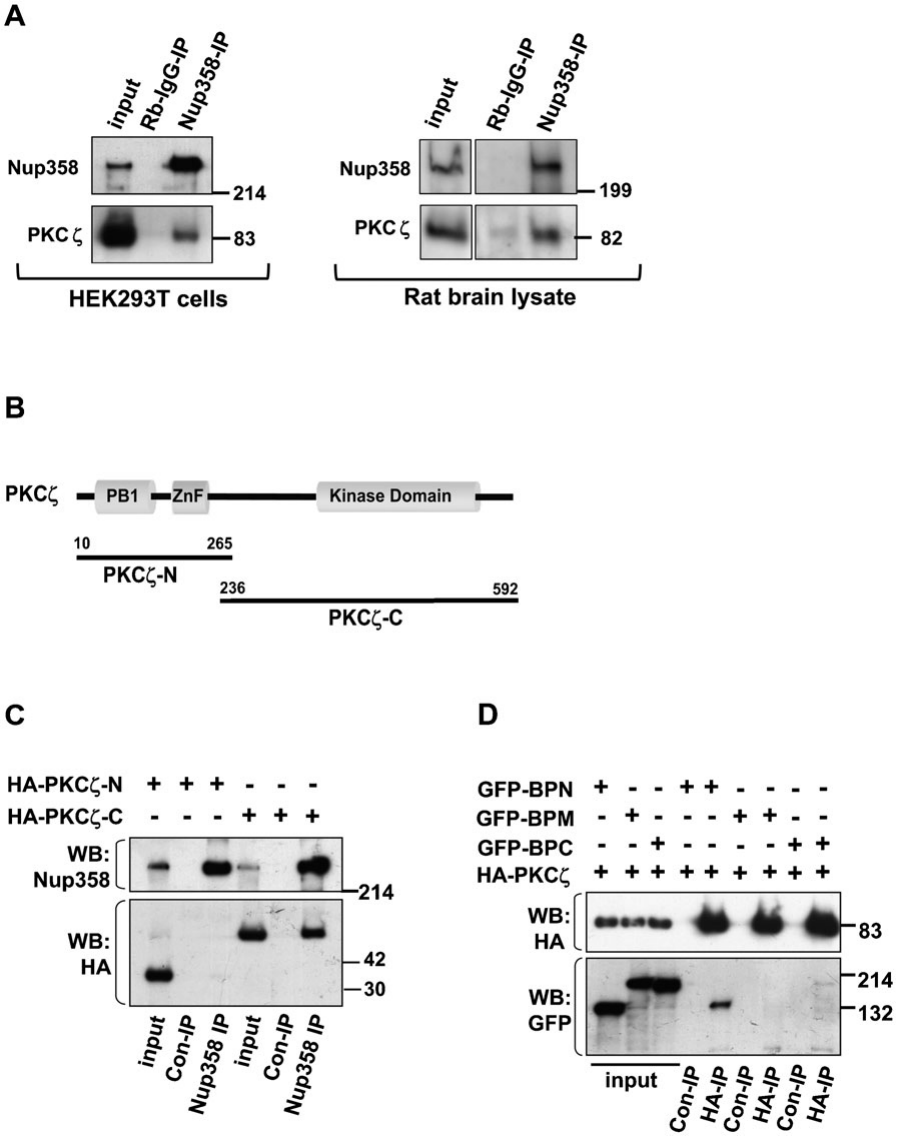
Nup358 interacts with aPKC. (A) *Left panel*: HEK293T was subjected to immunoprecipitation using rabbit polyclonal antibodies against Nup358 (Nup358-IP) or control rabbit IgG (Rb-IgG-IP), and the immunoprecipitates were immunoblotted using indicated antibodies. *Right panel:* E18 rat brain lysate was subjected to immunoprecipitation using Nup358 antibody. The immunoprecipitate was probed with aPKC (PKCζ) and Nup358 antibodies. (B) The domain structure of PKCζ and the fragments used in the study. (C) Lysates of HEK293T cells expressing HA-tagged fragments of PKCζ were subjected to immunoprecipitation using control Rb-IgG (Rb-IgG-IP) or anti-Nup358 (Nup358-IP) antibodies. The immunoprecipitates were probed with HA-specific antibodies and MAb414 (for Nup358). (D) Cells were co-transfected with HA-PKCζ and GFP-tagged version of BPN, BPM or BPC, and the lysates were subjected to immunoprecipitation using EZview control (Con-IP) or EZview HA (HA-IP) beads (Sigma–Aldrich). Immunoblotting was performed using indicated antibodies.

As previous studies in migrating astrocytes and hippocampal neurons have suggested that Dvl acts through regulation of aPKC (Schlessinger et al., 2007; Zhang et al., 2007), we were interested to investigate if any physical association and/or functional connection exist between Nup358 and aPKC. The subgroup of aPKCs in mammalian cells comprises three members, PKCι/λ, PKCζ, and PKMζ. Immunoprecipitation of endogenous Nup358 and western analysis of the immunoprecipitates with PKCζ antibodies, clearly showed that Nup358 and aPKC interact with each other *in vivo* (Fig. 2A). Also, we observed that the kinase activity of PKCζ was not essential for the interaction with Nup358, since similar extent of Nup358 interaction was observed with wild type as well as a kinase dead (KD) mutant of PKCζ (K238R) (supplementary material Fig. S2A). Nup358 was also found to interact with ectopically expressed HA-Par3 and myc-Par6, indicating that this nucleoporin could associate with Par complex *in vivo* (supplementary material Fig. S2B,C).

We further identified the domains of PKCζ and Nup358 important for the interaction with each other by immunoprecipitation assays. The results suggested that the C-terminal region of PKCζ, consisting of the catalytic domain (Fig. 2B,C), and N-terminal region of Nup358 (BPN, Fig. 2D) are involved in the interaction. We observed that PKCζ also associated with BPC to a much lesser extent (Fig. 2D, last lane).

### Downregulation of Nup358 leads to generation of multiple axons and overexpression of BPN inhibits axon–dendrite differentiation

**Fig. 3.**
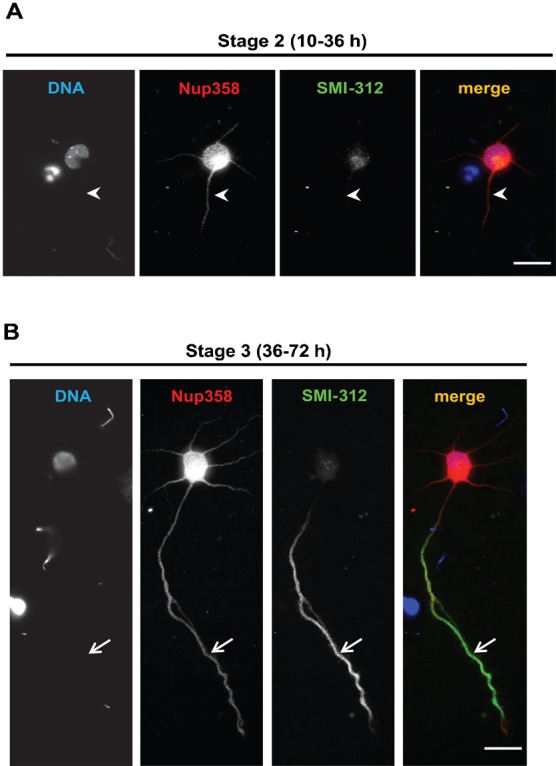
Localization of endogenous Nup358 in polarizing rat hippocampal neurons. Cultured rat hippocampal neurons, at stage 2 (A) and stage 3 (B), were immunostained with Nup358 (red) and SMI-312 (axonal marker; green). Arrow heads indicate immature neurites at stage 2 (B). Arrows indicate axons as identified SMI-312 axonal marker. DNA was visualized by Hoechst 33342 staining (blue). Scale bar, 25 µm.

To investigate the functional significance of interaction of Nup358 with Dvl1 and aPKC in cell polarization, we studied the process of axon–dendrite differentiation in cultured hippocampal neurons. During initial stages of polarization, the neurons proceed through several distinct stages (Arimura and Kaibuchi, 2007). After plating of hippocampal neuronal cells *in vitro*, within 10–36 hours, the cells develop short neurites of almost equal lengths that express no axonal marker (stage 2). Out of these, one neurite grows exponentially and forms an axon within 36–72 hours (stage 3). We analyzed the localization of endogenous Nup358 in differentiating neurons. In addition to the presence at the nuclear pore complex, Nup358 also showed a uniform localization in the soma of neurons and in all neurites at stage 2 (Fig. 3A, arrow head). At stage 3, Nup358 was more or less evenly distributed throughout the neuron including axon and dendrites, whereas the axonal marker SMI-312 accumulated exclusively in the axon (Fig. 3B, arrow).

**Fig. 4.**
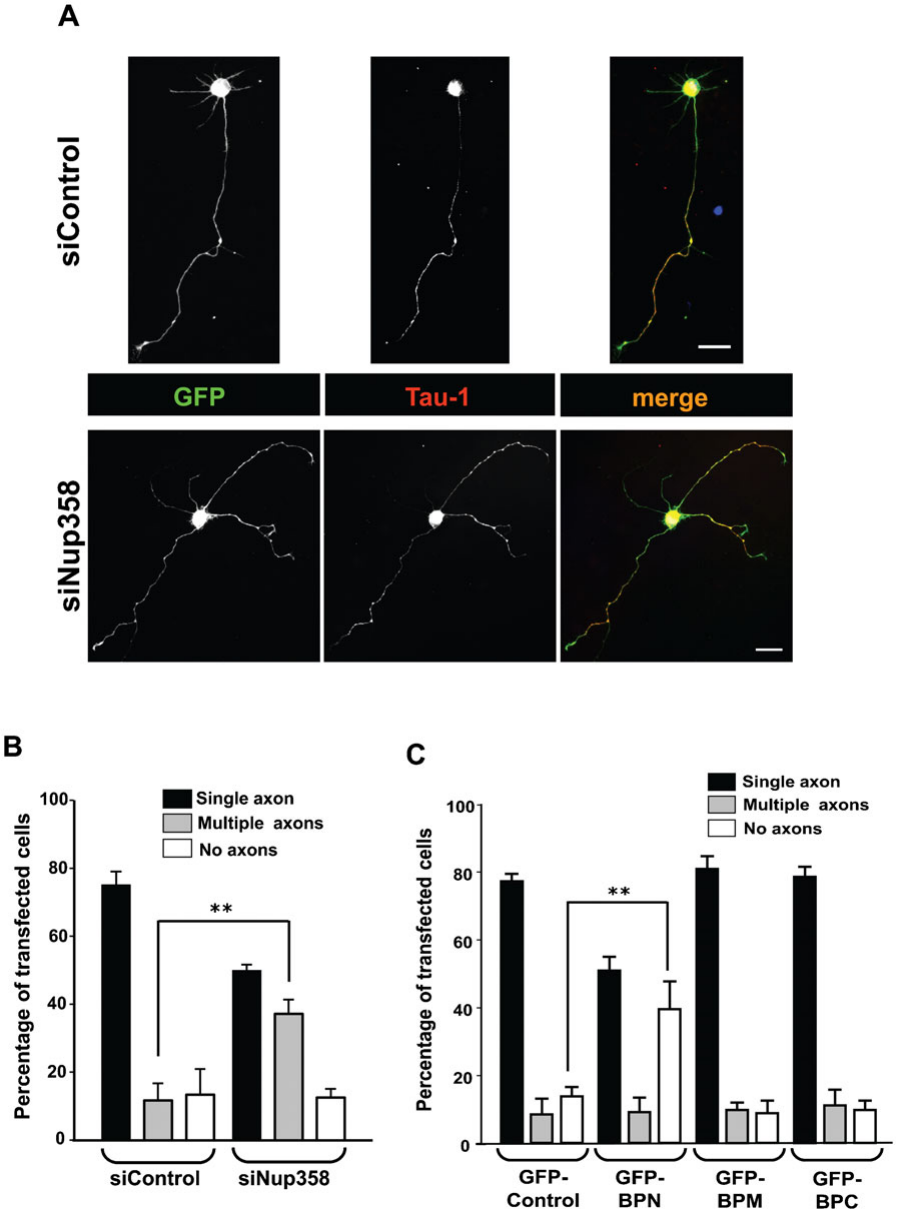
Nup358 is required for neuronal polarization. (A) E18 hippocampal neurons were transfected with control (siControl) or Nup358 siRNA (siNup358) along with pBetaActin-eGFP as transfection control, and were immunostained after culturing for 72 hours *in vitro*. The transfected neurons were identified by GFP expression (green). The effect of Nup358 depletion on axon formation was analysed using anti-Tau-1 antibodies (red). Arrows indicate axons as identified by Tau-1 axonal marker. DNA was stained with Hoechst 33342 dye (blue). Scale bar, 25 µm. (B) Quantitative analysis of the effect of Nup358 depletion on neuronal polarity. Error bars indicate standard deviations, *n* = 3, **P<0.01, Student's *t* test. (C) E18 rat hippocampal neurons were transfected with GFP-control, GFP tagged version of BPN, BPM or BPC and assessed for the effect on neuronal polarization after 72 hours. Error bars indicate standard deviations, *n* = 3, **P<0.01, Student's *t* test.

To determine whether Nup358 plays a role in the process of axon specification *in vitro*, we resorted to siRNA-mediated depletion of Nup358 in hippocampal neurons isolated from E18 rat embryos. Nup358 siRNA or control siRNA was transfected into hippocampal neurons along with pBetaActin-eGFP using Amaxa nucleofector. The neurons were cultured for 72 hours and assessed for axon–dendrite differentiation by immunostaining with specific antibodies against Tau-1 (axonal marker). Majority of the control siRNA transfected cells produced single axons. However, upon Nup358 depletion, a significantly higher number of GFP-positive (transfected) cells developed multiple axons (more than one axon), as compared to the control siRNA treated cells (Fig. 4A,B). Furthermore, an independent siRNA targeted against rat Nup358 (siNup358 no. 2) also showed significant increase in multiple axon formation as compared to the control siRNA (supplementary material Fig. S3A). Moreover, expression of human Nup358, which is resistant to the siRNA directed against rat Nup358 due to sequence variation in the siRNA binding site, could significantly reverse the multiple axon phenotype produced by depletion of rat Nup358 (supplementary material Fig. S3B). To validate the efficiency of Nup358 depletion, we subjected H9c2, a rat cardiomyocyte cell line, to Nup358 siRNA treatment and assessed the endogenous levels of Nup358 using immunofluorescence and western blot analysis (supplementary material Fig. S4A,B). A significant reduction in Nup358 level was also observed in rat hippocampal neurons transfected with Nup358 siRNA as compared to control siRNA treated cells, using immunofluorescence microscopy (supplementary material Fig. S4C). We also assessed the axon–dendrite polarization under control or Nup358 siRNA treated condition by staining for different neuronal markers, MAP2 (dendrite-specific) and SMI-312 (axon-specific) (supplementary material Fig. S5).

Further studies were performed to determine the effect of overexpression of different fragments of Nup358 on the establishment of neuronal polarity. We observed that a significantly higher number of the cells transfected with BPN did not develop axons, whereas, overexpression of BPM and BPC did not affect the axon–dendrite polarization (Fig. 4C). Together, these results suggest that Nup358 plays an important role in neuronal polarization.

### Multiple axon phenotype generated by Nup358 depletion is partially reversed by Dvl1 depletion

Previous studies have shown that ectopic expression of Dvl1 results in the formation of multiple axons, whereas, its downregulation leads to no axon formation (Zhang et al., 2007). Therefore, we wished to test whether Dvl and Nup358 are a part of the same polarity cascade, and if so, which one acts upstream of the other. We confirmed the *in vivo* interactions of Nup358 with Dvl1 in cultured primary neurons (Fig. 1A right panel).

**Fig. 5.**
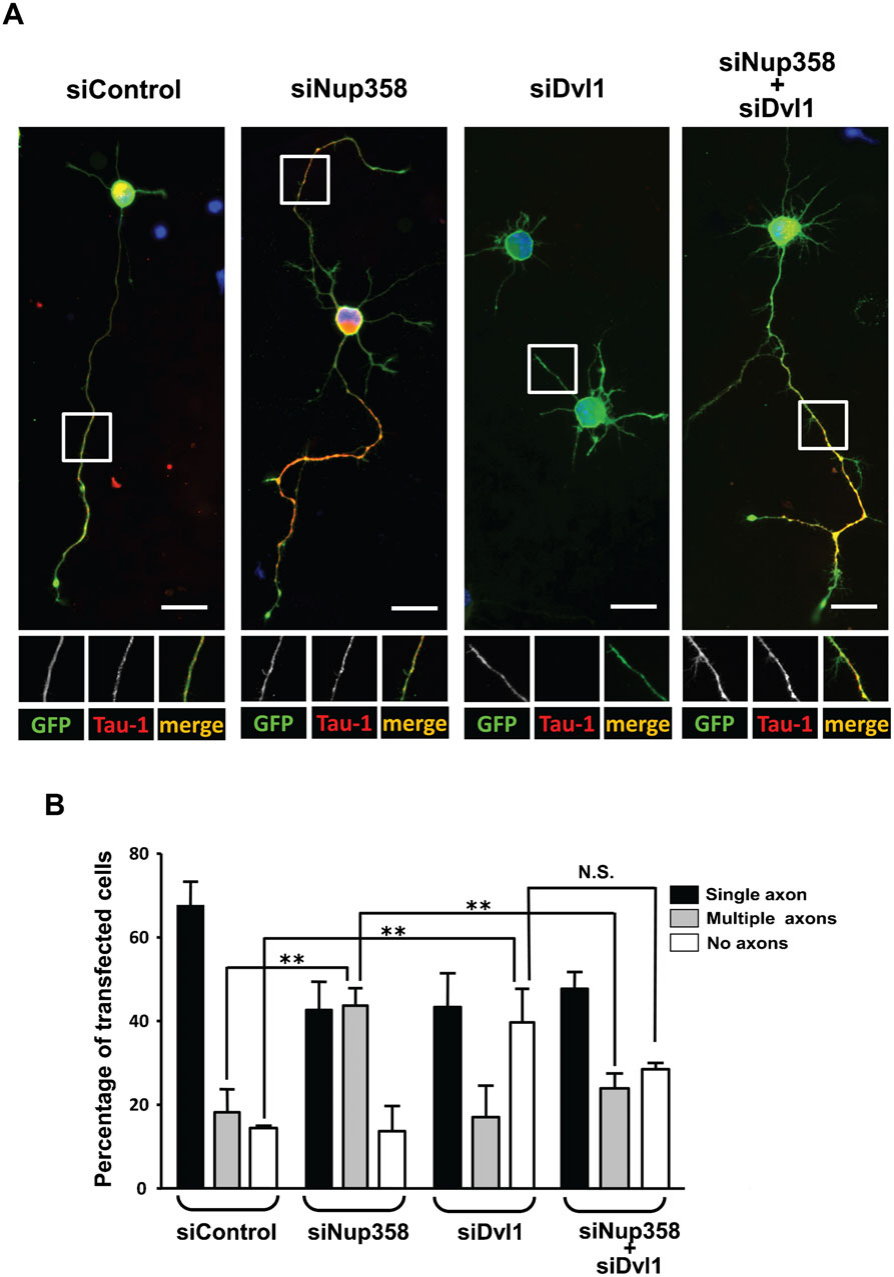
Nup358 functions upstream of Dvl. (A) Hippocampal neurons were transfected with control siRNA (siControl), Nup358 siRNA (siNup358), Dvl1 siRNA (siDvl1) alone or co-transfected with siNup358 and siDvl1 as indicated. pBetaActin-eGFP (green) was used as the transfection marker. The transfected cells were stained with Tau-1 (red) to study the effect on axon formation. DNA was stained with Hoechst 33342 dye (blue). Scale bar, 25 µm. (B) Quantitative analysis of the effect of siRNA mediated depletion of different proteins on axon formation. Error bars indicate standard deviations, *n* = 3, **P<0.01, N.S. – non-significant, Student's *t* test.

To explore the functional relationship between Nup358 and Dvl1, co-transfection studies using siRNAs against Nup358 and Dvl were performed. The results indicated that the multiple axons phenotype generated by Nup358 depletion could be significantly reversed by simultaneous depletion of Dvl from developing neurons (Fig. 5A,B). Based on these results, we conclude that Nup358 acts upstream of Dvl in the molecular cascade responsible for generation of polarity in neurons.

### Multiple axon phenotype produced by Nup358 depletion is partially reversed by inhibition of aPKC activity

**Fig. 6.**
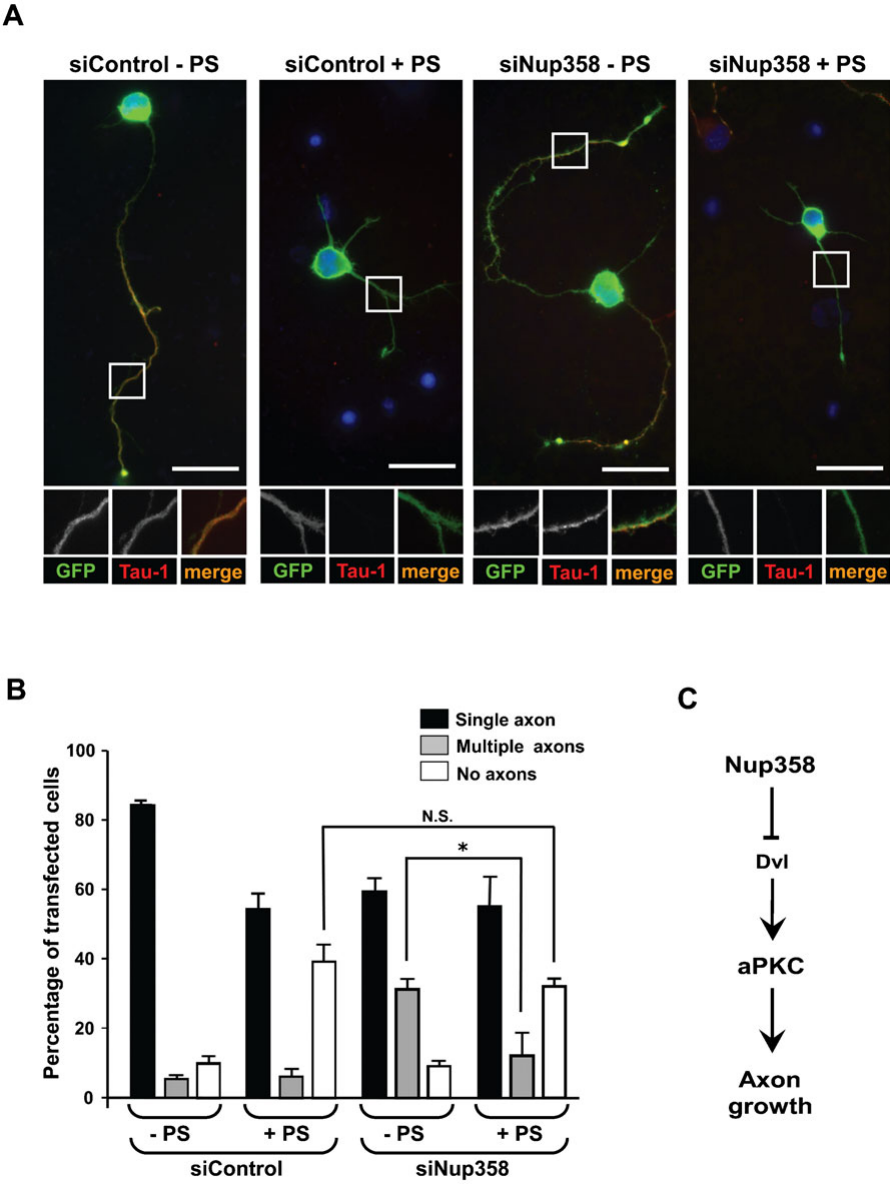
Multiple axons formed by Nup358 depletion is partially rescued by inhibition of aPKC activity. (A) Hippocampal neurons transfected with control (siControl) or Nup358 (siNup358) siRNA along with pBetaActin-eGFP and were cultured in the presence (+ PS) or absence (− PS) a pseudosubstrate inhibitor specific for PKCζ (10 µM). The neurons were fixed and immunostained after 72 hours. The effect of different treatments on neuronal polarization was assessed by scoring for GFP expression (green) and Tau-1 staining (red). Scale bar, 25 µm. (B) The quantitative analysis of the effect on neuronal polarity. Error bars indicate standard deviations, *n* = 3, *P<0.05, Student's *t* test. (C) A working model for the function of Nup358 in neuronal polarization. During the initial stages of single axon formation, Dvl-mediated activation of aPKC occurs at the nascent axon. Differentiation of other neurites into axons could be spatially and temporally inhibited by Nup358-mediated interference of Dvl and aPKC functions.

It has been shown that recruitment of Par complex and localized activation of aPKC at the nascent axon is instrumental in breaking the symmetry and enhancing the axon growth (Arimura and Kaibuchi, 2007; Shi et al., 2003). However, the aPKC activity needs to be inhibited at other neurites to subsequently achieve the axon–dendrite polarity. We confirmed the *in vivo* interactions of Nup358 with aPKC in neurons by immunoprecipitation assay using rat brain lysate (Fig. 2A right panel). Since depletion of Nup358 results in the generation of multiple axons, one possible explanation could be that Nup358 negatively regulates axon formation by inhibition of aPKC at the neurites that eventually differentiate into the dendrites. To test this hypothesis, Nup358 siRNA transfected neurons were cultured in the presence or absence of aPKC inhibitor, and assessed for the effect on neuronal polarization. In the presence of aPKC inhibitor, a significant number of cells displayed no axon phenotype as reported earlier (Shi et al., 2003), which remained almost unaffected under conditions of control or Nup358-specific siRNA treatment. However, the multiple axon phenotype produced by Nup358 depletion was significantly reduced when the cells were treated with the aPKC inhibitor (Fig. 6A,B). This indicates that Nup358 could negatively regulate aPKC function.

## Discussion

Our studies indicate a conserved role of the nucleoporin Nup358 in the establishment of cell polarity. Depletion of Nup358 from developing neurons led to aberrant axon–dendrite polarization and multiple axon formation. Further analysis suggested that Nup358 acts upstream of Dvl1 and aPKC in the molecular cascade that regulates neuronal polarity. However, understanding the molecular details of how Nup358 functions in neuronal polarization in coordination with Dvl and aPKC requires further investigation. As initial stages of axon specification requires multiple negative and positive feed-back loops operating in a concerted manner, and ultimately assuring that one among the many neurites develops into an axon (Arimura and Kaibuchi, 2007; Barnes and Polleux, 2009), we speculate that Nup358 would inhibit differentiation of other neurites into axons by negatively regulating the function of Dvl and aPKC (Fig. 6C). It is possible that Wnt-mediated activation of Dvl could overcome the inhibition caused by Nup358 in the developing axon. Further studies are required for testing this hypothesis. Interestingly, Dvl1 and aPKC localize to the tip of most neurites at stage 2, and preferentially accumulate on axons and axonal tips at stage 3 (Zhang et al., 2007). Nup358, however, showed uniform distribution in the soma of neurons at stage 2, and axon and dendrites at stage 3 (Fig. 3). The differential and reciprocal localization of Dvl1/aPKC and Nup358 could support the functional antagonism existing between them in coordinating the polarization events during axon–dendrite differentiation.

How does Nup358 regulate the activity of aPKC is still an open question. It is possible that Nup358 could interfere with the formation of functional Par complex. In fact, Nup358 interacts with the C-terminal region of PKCζ, a region already shown to be involved in the interaction of aPKC with Par3 and Dvl1 (Izumi et al., 1998; Zhang et al., 2007). Interestingly, expression of a fragment of Nup358 (BPN), which interacts with aPKC in developing neurons led to no axon formation (Fig. 2). It remains to be investigated if Nup358 association affects the interaction of aPKC with other known interacting partners.

Nup358 is a Ran binding protein and also acts as a SUMO E3 ligase (Pichler et al., 2002; Wu et al., 1995; Yokoyama et al., 1995). It is possible that these activities contribute to its function in neuronal polarization. Interestingly, previous studies have identified a cytoplasmic role for RanGTPase in retrograde injury signalling in peripheral nerves (Yudin et al., 2008; Yudin and Fainzilber, 2009). As Nup358 exists in complex with SUMO-modified RanGAP1, and this complex is assumed to be responsible for GTP hydrolysis on Ran, removal of Nup358 would be predicted to decrease the RanGAP1 activity, and thereby leading to the accumulation of RanGTP in the cytoplasm. It remains to be seen if Nup358 functions in neuronal polarity through regulating the cytoplasmic RanGTP levels.

Nup358 could also affect the dynamics of the microtubule cytoskeleton (Joseph and Dasso, 2008), which is a key determinant of axon–dendrite polarization (Conde and Cáceres, 2009). Our study indicates the possibility of Nup358 coupling the regulation of microtubule dynamics to the activities of Dvl and Par polarity proteins. Based on the results presented, we conclude that Nup358 plays a conserved role in cell polarization by functionally interacting with the components of Wnt and Par polarity pathways. Further studies are required to unravel the molecular details of how Nup358 functions in the establishment of polarity.

Recently, in addition to the nucleo-cytoplasmic transport, many nucleoporins have been known to play critical roles in diverse cellular processes such as mitosis, gene transcription, chromatin remodelling and cell migration (D'Angelo and Hetzer, 2008). Our finding that Nup358 plays a crucial role in neuronal polarization adds to the growing list of non-traditional functions of nucleoporins.

## Materials and Methods

### Reagents and constructs

The antibodies used were: mouse anti-PKCζ (H-1), mouse anti-Dvl1 (3F12), mouse anti-GFP (B-2), mouse anti-c-myc (9E10) from Santa Cruz Biotechnology, rabbit IgG from Bangalore Genei/Vector Laboratories, mouse anti-FxFG-containing nucleoporins (mAb414), mouse anti-SMI-312 and mouse anti-HA from Covance, mouse anti-Tau-1 and mouse anti-MAP2 from Chemicon/Millipore. Rabbit polyclonal antibodies against Nup358 and GFP were described previously (Joseph et al., 2004; Sahoo et al., 2012). Antibodies against recombinant His-tagged mouse Dvl1 C-terminus (554–695 amino acids) was raised and affinity-purified as described earlier (Sahoo et al., 2012). This antibody was used for immunoprecipitating endogenous Dvl1 from E18 Wistar rat cultured primary neurons.

GFP-tagged versions of full-length and fragments of human Nup358 (GFP-BPN, GFP-BPM and GFP-BPC) have been described earlier (Joseph and Dasso, 2008). Mouse Dvl1 was PCR amplified from pcDNA-GFP-mDvl1 (kindly provided by Lin Li, Shanghai Institutes for Biological Sciences, China) and cloned into *Eco*RI-*Sma*I sites of pEGFP-C2 to generate pEGFP-Dvl1. The fragments of Dvl1 [Dvl1-N (2–210 amino acids), Dvl-M (211–425 amino acids), Dvl-C (426–695 amino acids)] were PCR amplified from pEGFP-Dvl1 and cloned into *Eco*RI-*Sma*I sites of pEGFP-C2. Mouse Dvl1ΔPDZ was generated by deleting the region between 211 and 425 amino acids by PCR based method. For constitutive expression in neurons (Fig. 2), BPN and Dvl1 were subcloned into pBetaActin-eGFP (kindly provided by Gary Banker, Oregon Health Science University, USA). BPN fragment was cloned at *Hin*dIII and StuI sites of pBetaActin-eGFP to produce BPN with GFP fused at its C terminus. mDvl1 was subcloned from pcDNA-HA-Dvl1 into pBetaActin-eGFP at *Hin*dIII and XbaI sites to generate HA-tagged Dvl1 construct. Note that *Hin*dIII-XbaI digestion removed GFP from pBetaActin-eGFP. Mouse Dvl2 and Dvl2 deletion mutants have been described previously (Sahoo et al., 2012).

The rat Par3 homolog ASIP was subcloned from SRHisB-rASIP (a kind gift from Shigeo Ohno, Yokohama City University School of Medicine, Japan) into pcDNA-HA-C1 at the EcoRV-Xho1 sites. pCAN-myc-hPAR6C, pCAN-HA-hPKCζ wild type (WT) and pCMV5-rPKCζ kinase dead (KD) mutant (K281W) were provided by Steven Martin (University of California, Berkeley, USA). PKCζ WT was subcloned into pEGFP-C2 (Clontech) using *Eco*RI and ApaI sites and the KD mutant was cloned into *Eco*RI site of pEGFP-C2. The deletion clones pcDNA-PKCζ-N (10–265 amino acids) and pcDNA-PKCζ-C (236–592 amino acids) were generated from pCAN-HA-hPKCζ by digestion with appropriate restriction enzymes and self-ligation.

pBetaActin-eGFP was used as transfection marker in hippocampal neuronal experiments. Myristoylated pseudosubstrate inhibitor of PKCζ was purchased from Sigma–Aldrich.

### Cell culture and transfections

HEK293T, COS-7 and H9c2 cells were cultured in DMEM supplemented with 10% Fetal Bovine Serum (FBS). The DNA constructs were transfected using Lipofectamine 2000 (Invitrogen) or polyethylene imine (Polysciences, Inc.), according to manufacturer's instructions.

Hippocampal neurons were isolated from E18 Wistar rats and were cultured as described previously (Kaech and Banker, 2006). Briefly, the hippocampi were trypsinized using TPVG (HiMedia Laboratories) for 30 minutes at 37°C and the dissociated cells were transfected with DNA constructs using Amaxa nucleofector (Lonza) according to manufacturer's instructions. The transfected cells were plated on 0.1% Poly-L-Lysine (Sigma–Aldrich)-coated coverslips, in a mixture of DMEM + HAM's F12 (1:1), 10% FBS, 5% horse serum, 0.5% Glutamax (Invitrogen) and 5 µg/ml Ciplox (Cipla Ltd). After 6 hours of plating, cells were grown in Neurobasal medium (Invitrogen) supplemented with 2% B27 (Invitrogen), 5 µg/ml Ciplox and 1% Glutamax. Neuronal morphology was analysed by immunostaining after 72 hours of plating. All animal handling and experimental procedures were approved by the National Centre for Cell Science Institutional Animal Ethics Committee.

The sequences for control and Nup358 siRNAs have been reported earlier (Murawala et al., 2009), and the targeting sequence for Nup358 is conserved in rat as well. This has been named siNup358 in the paper and siNup358 no. 1 in some places as indicated. An additional siRNA designed specifically against rat Nup358 (5′-GGAAGGCGAGTGGGAGTGT-3′) was also used to deplete Nup358 (supplementary material Fig. S3). This has been referred to as siNup358 no. 2, wherever relevant. The sequence used for the siRNA against rat Dvl1 has been described earlier (Zhang et al., 2007). All the siRNAs were obtained from Dharmacon/Thermo Scientific. Transfection of siRNAs and DNA constructs in hippocampal neurons was performed with Amaxa nucleofector II (Lonza), and pBetaActin-eGFP was used as the transfection marker. H9c2 cells were transfected with siRNAs using Lipofectamine 2000 (Invitrogen) and were analyzed for Nup358 depletion after 72 hours.

### Immunostaining

For immunofluorescence analysis, COS-7 cells were fixed 24–36 hours after transfection with 100% chilled methanol for 5 minutes and were given a quick wash with 0.1% Triton X-100. Primary and secondary antibody incubations were performed in 2% normal horse serum (NHS, Vector Laboratories) in 1× TBS. Hoechst 33342 dye (Sigma–Aldrich) was used for visualization of nucleus.

Hippocampal neurons were fixed in 1× PBS containing 4% PFA and 4% sucrose for 20 minutes, followed by permeabilization by 0.2% Triton X-100 for 10 minutes. Primary and secondary antibody incubations were performed in 1× PBS containing 1% NHS and 1% BSA for 45–90 minutes. The antibodies and dilutions used were: rabbit polyclonal anti-Nup358 (1:500), mouse anti-HA (1:200), mouse monoclonal anti-Tau-1 (1:1000), mouse monoclonal anti-SMI-312 (1:3000) and mouse anti-MAP2 (1:50). Fluorescently labelled secondary antibodies (Alexa 488, Alexa 568 or 594; Invitrogen) were used at 1:1000 dilution. Images were obtained with an inverted fluorescence microscope (Axiovert 200M, Zeiss) using a Plan Apochromat 40× (1.3 NA) or 63× (1.4 NA) oil immersion objective. Projection images were generated from optical sections 100 nm apart, having a section thickness of 700 nm, using the Axiovision Extended Focus program. Images for Fig. 1E were obtained using a laser-scanning confocal microscope (TCS SP5; Leica). All the images were further processed in Adobe Photoshop CS3.

The transfected neurons were scored on the basis of the following criteria: i) neurons with short neurites around the cell body, which were of similar length and Tau-1 negative, were termed as no axons, ii) neurons with a single long neurite, which was Tau-1 positive and at least two times longer than the other neurites around the cell body, were termed as single axon, and iii) cells having two or more neurites, which were Tau-1 positive and at least two times longer than other neurites, were termed as multiple axons.

### Immunoprecipitation and western blotting

For immunoprecipitation analysis, the cells were washed once with ice cold 1× TBS and lysed in cytoskeleton extraction buffer (10 mM Tris-HCl pH 7.4, 100 mM NaCl, 1 mM EDTA, 1 mM EGTA, 1 mM NaF, 2 mM Na_3_VO_4_, 1% Triton X-100, 10% glycerol, 0.1% SDS, 0.5% deoxycholate) containing mammalian protease inhibitor cocktail (Roche Applied Science). After centrifugation at 15,294 *g* at 4°C for 30 minutes, the supernatant was incubated with protein A/G Sepharose beads (Santa Cruz Biotechnology) pre-bound with control IgG or indicated specific antibody. For HA immunoprecipitations, EZview Red control or anti-HA beads (Sigma–Aldrich) were used. After incubation for 2 hours at 4°C, the beads were washed three times with the lysis buffer, and the immunoprecipitates were eluted using 3× Laemmli sample buffer and were subjected to SDS-PAGE and western blotting using indicated antibodies.

For endogenous immunoprecipitations from neurons, cultured rat primary neurons from E18 rat (Fig. 1) or E18 rat brains (Fig. 2) were used, as indicated. The rat brains were isolated and crushed into fine powder in liquid nitrogen. The lysates for immunoprecipitation were prepared using NP-40 lysis buffer (20 mM Tris HCl pH 8, 137 mM NaCl, 10% glycerol, 1% nonidet P-40, 2 mM EDTA, 1 mM NaF, 2 mM Na_3_VO_4_) containing mammalian protease inhibitor cocktail (Roche Applied Science) and 200 mM PMSF. The lysate was subjected to sonication and ultra-centrifugation at 55,000 rpm in MLA-130 rotor (Beckman Coulter) for 45 minutes. The supernatant was used for immunoprecipitations with control rabbit IgG, Nup358 or Dvl1 antibodies. The immunoprecipitates were washed twice with lysis buffer and once with 1× TBS before proceeding for western analysis.

### Statistical analysis

All values are indicated as mean ± s.d. of three independent experiments. In each experiment, 80–100 cells were counted. *P* values were calculated using Student's *t* test and values less than 0.05 was considered as statistically significant.

## Supplementary Material

Supplementary Material
