# Supplementary material for: Nup358 interacts with Dishevelled and aPKC to regulate neuronal polarity
Source: Biol Open. 2013 Oct 16;2(11):1270–8. doi: 10.1242/bio.20135363 (PMC3828775; doi:10.1242/bio.20135363)
Supplement: Supplementary Material [file supp_2_11_1270__index.html]

Nup358 interacts with Dishevelled and aPKC to regulate neuronal polarity — Supplementary Material 

# Nup358 interacts with Dishevelled and aPKC to regulate neuronal polarity

## bio.20135363 Supplementary Material

**Files in this Data Supplement:**

- Supplementary Material - Pankhuri Vyas et al. doi: 10.1242/bio.20135363
