# Supplementary material for: Nup358 interacts with Dishevelled and aPKC to regulate neuronal polarity
Source: Biol Open. 2013 Oct 16;2(11):1270–8. doi: 10.1242/bio.20135363 (PMC3828775; doi:10.1242/bio.20135363)
Supplement: Supplementary Material [file supp_bio.20135363_bio.20135363-s1.pdf]

Biology Open

Supplementary Material

Pankhuri Vyas et al. doi: 10.1242/bio.20135363

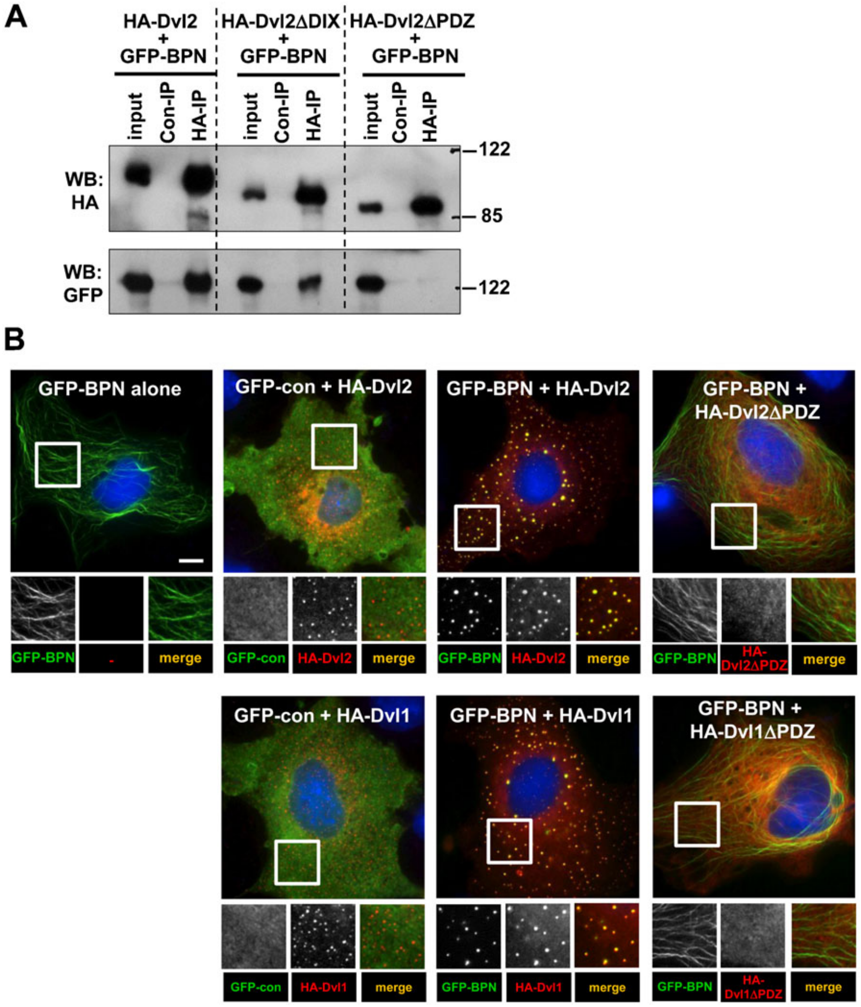

Fig. S1. The N-terminal region of Nup358 (BPN) interacts with Dvl in a PDZ dependent manner. (A) HEK293T cells were co-transfected HA-Dvl2, HA-Dvl2ΔDIX or HA-Dvl2ΔPDZ with GFP-BPN and were subjected to immunoprecipitation with control protein A beads (Con-IP) or HA-antibody bound protein A beads (HA-IP). The immunoprecipitates were probed with indicated antibodies by western blotting (WB). (B) COS-7 cells were transfected with GFP (GFP-con) or GFP-BPN and HA-Dvl2, HA-Dvl2ΔPDZ, HA-Dvl1 or HA-Dvl1ΔPDZ as indicated. GFP is visualized by epifluorescence (green) and HA tagged proteins were detected using HA-specific antibodies (red). DNA is stained with Hoechst 33342 dye (blue).

Biology Open

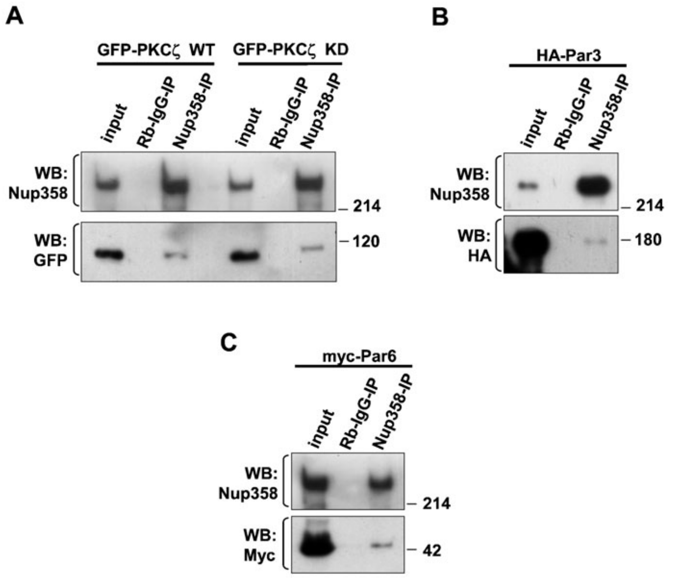

Fig. S2. Interaction of Nup358 with components of Par polarity complex. (A) Nup358 interacts with PKCζ independent of its kinase activity. GFP-tagged PKCζ wild type (WT) and Kinase dead mutant (KD) were overexpressed in HEK293T cells, and immunoprecipitation of endogenous Nup358 was performed using anti-Nup358 antibodies (Nup358-IP), while rabbit-IgG (Rb-IgG-IP) was used as control. Western blotting (WB) was done using anti-GFP and mAb414 (for Nup358) antibodies. Nup358 interacts with PAR3 (B) and Par6 (C). HA-PAR3 or myc-PAR6 was overexpressed in HEK293T cells and immunoprecipitation was performed using anti-Nup358 antibodies (Nup358-IP). Rabbit IgG was used as control (Rb-IgG-IP). The immunoprecipitates were probed with indicated antibodies.

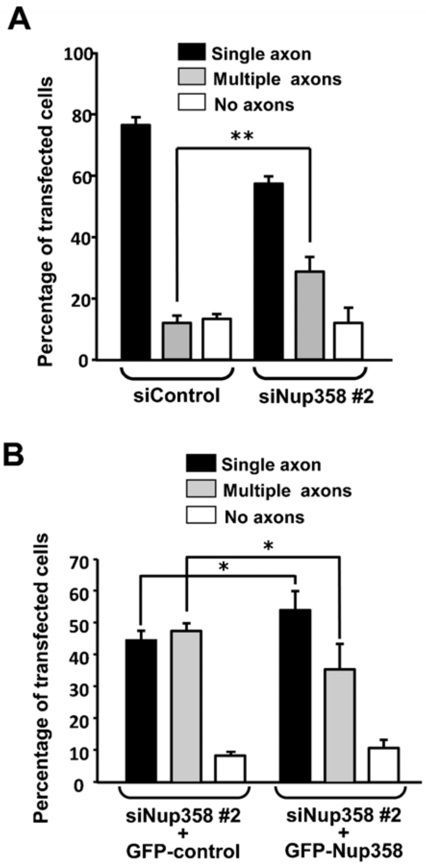

Fig. S3. Overexpression of Nup358 significantly rescues phenotypes caused due to Nup358 depletion in rat hippocampal neurons. (A) Quantitative data representing polarization of rat hippocampal neurons transfected with control siRNA (siControl) or specific siRNA against rat Nup358 (siNup358 no. 2). Error bars indicate standard deviations, $n=3$, $^{**}P<0.001$, Student's t test. (B) Rat hippocampal neurons were transfected with siNup358 no. 2 and GFP control or GFP-Nup358 constructs as indicated. Neuronal polarization of transfected cells was assessed by Tau-1 staining. Error bars indicate standard deviations, $n=4$, $^{*}P<0.05$, Student's t test.

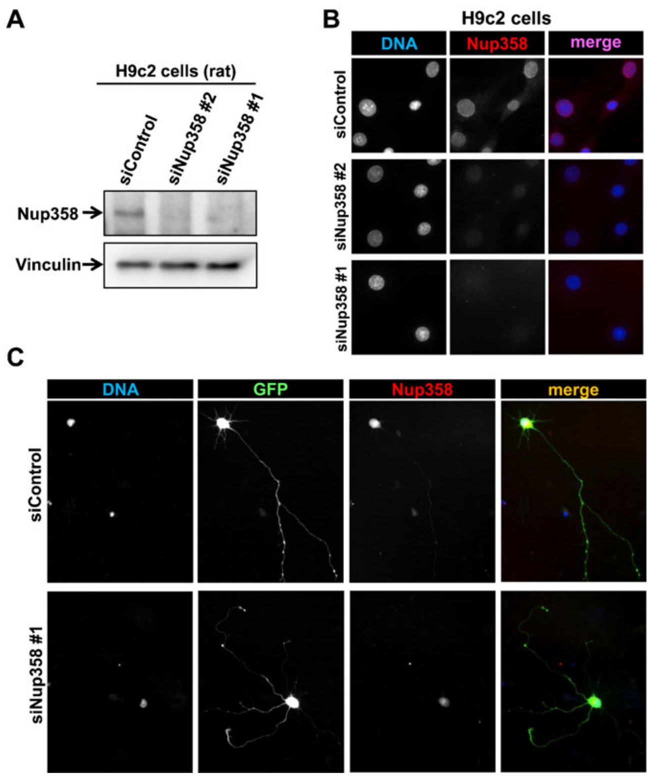

Fig. S4. Nup358 depletion leads to multiple axon formation. (A) Rat cardiomyocyte cells (H9c2) were transfected with control siRNA (siControl) or individual Nup358 specific siRNA (siNup358 no. 1 or no. 2). siNup358 no. 1; the siRNA used throughout the paper, and named siNup358 elsewhere. siNup358 no. 2; rat specific siRNA against Nup358 (target sequence: 5′-GGAAGGCGAGTGGGAGTGT-3′) and were subjected to western blotting using Nup358 antibody. Vinculin was used as loading control. (B) H9c2 cells from the above-mentioned experiments were fixed and stained for Nup358 with specific antibodies (red). (C) Rat hippocampal neurons transfected with control (siControl) or Nup358 specific siRNA (siNup358 no. 1) and pBetaActin-eGFP (as transfection marker, GFP, green) were stained for Nup358 (red). DNA was stained with Hoechst 33342 dye (blue).

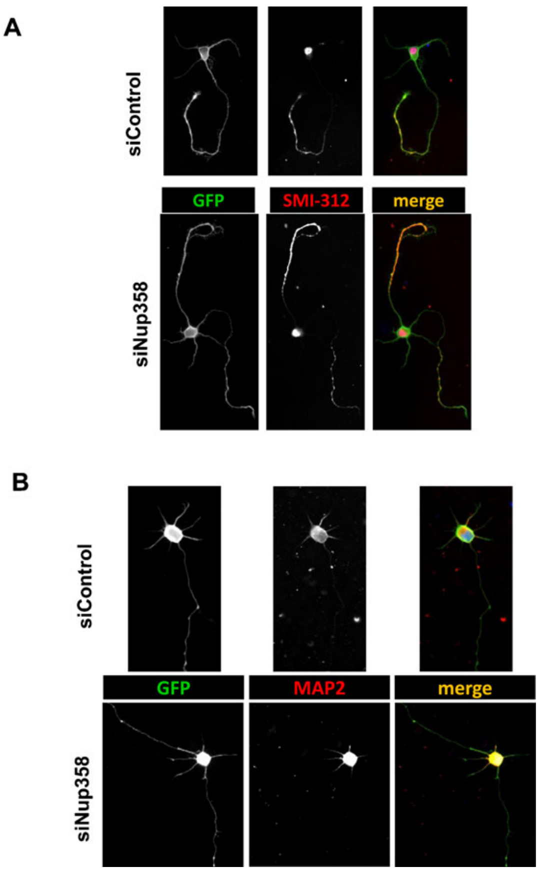

Fig. S5. Nup358 depletion leads to multiple axon formation. Rat hippocampal neurons transfected with control siRNA (siControl) or Nup358 siRNA (siNup358) and pBetaActin-eGFP (as transfection marker, GFP, green) were stained with axonal marker SMI-312 (A, red) or dendrite marker MAP2 (B, red). DNA is visualized by Hoechst 33342 staining (blue).
